# Full-Length Multi-Barcoding: DNA Barcoding from Single Ingredient to Complex Mixtures

**DOI:** 10.3390/genes10050343

**Published:** 2019-05-07

**Authors:** Peng Zhang, Chunsheng Liu, Xiasheng Zheng, Lan Wu, Zhixiang Liu, Baosheng Liao, Yuhua Shi, Xiwen Li, Jiang Xu, Shilin Chen

**Affiliations:** 1 School of Chinese Materia Medica, Beijing University of Chinese Medicine, Beijing 102488, China; pzhang@icmm.ac.cn (P.Z.); max_liucs@263.net (C.L.); 2 Key Laboratory of Beijing for Identification and Safety Evaluation of Chinese Medicine, Institute of Chinese Materia Medica, China Academy of Chinese Medical Sciences, Beijing 100700, China; lwu@icmm.ac.cn (L.W.); zhixiangliu88@163.com (Z.L.); swjs082lbs@126.com (B.L.); yhshi@icmm.ac.cn (Y.S.); xwli@icmm.ac.cn (X.L.); 3 Guangdong Provincial Key Laboratory of New Drug Development and Research of Chinese Medicine, Guangzhou University of Chinese Medicine, Guangzhou 510006, China; zheng.x.s1987@163.com

**Keywords:** DNA barcoding, single-molecule real-time (SMRT), multi-species mixtures, next-generation sequencing, Sheng-Mai-San (SMS), ITS2

## Abstract

DNA barcoding has been used for decades, although it has mostly been applied to some single-species. Traditional Chinese medicine (TCM), which is mainly used in the form of combination-one type of the multi-species, identification is crucial for clinical usage. Next-generation Sequencing (NGS) has been used to address this authentication issue for the past few years, but conventional NGS technology is hampered in application due to its short sequencing reads and systematic errors. Here, a novel method, Full-length multi-barcoding (FLMB) via long-read sequencing, is employed for the identification of biological compositions in herbal compound formulas in adequate and well controlled studies. By directly sequencing the full-length amplicons of ITS2 and *psbA-trnH* through single-molecule real-time (SMRT) technology, the biological composition of a classical prescription *Sheng-Mai-San* (SMS) was analyzed. At the same time, clone-dependent Sanger sequencing was carried out as a parallel control. Further, another formula—*Sanwei-Jili-San* (SJS)—was analyzed with genes of ITS2 and CO1. All the ingredients in the samples of SMS and SJS were successfully authenticated at the species level, and 11 exogenous species were also checked, some of which were considered as common contaminations in these products. Methodology analysis demonstrated that this method was sensitive, accurate and reliable. FLMB, a superior but feasible approach for the identification of biological complex mixture, was established and elucidated, which shows perfect interpretation for DNA barcoding that could lead its application in multi-species mixtures.

## 1. Introduction

As DNA barcoding, a dominant method for species identification and discovery [1], emerges as a cost-effective standardized approach for rapid species identification [2,3], it has been widely used in almost all types of organisms. For traditional Chinese medicine (TCM), whose identification is crucial for its safety and effectiveness [4,5,6,7] in clinical practice, various methods have been applied, such as microscopic analysis, chromatography, spectroscopic methodology and molecular biology. Molecular biology methods, especially DNA barcoding, are relatively more precise and sensitive in general [8,9,10]. As a consequence, the DNA barcoding system for identifying herbal medicine (TcmBarcode system, http://www.tcmbarcode.cn/en/) [11] has been successfully established and widely used [12].

For Chinese herbal compounds, most of which are mixtures in the form of pills, powders or other dosage forms, the separation of different raw materials seems impossible, while parts of nucleic acid have been degraded, resulting in difficulties for biological identification [13]. Comparing with Sanger sequencing, in which separation and purification like cloning are commonly needed [14,15], next-generation sequencing (NGS) shows deeper sequencing depth and higher benefit-cost ratio [16] that has revealed legality issues and health safety concerns on TCM [17]. NGS, mainly second-generation sequencing, is considered a powerful approach for the analysis of biotic mixtures, such as the microorganism of the environment [18,19], the soil [20], the gut [21,22,23,24], and the food [25]. Several strategies, such as PCR-free metabarcoding [26], mass-PCR metabarcoding [27], multi-marker metabarcoding [28] and even metagenomics [29] have been employed to evaluate the biodiversity of different biotic communities. But some improvements are still needed, such as primers design [30], fractioning step [31] and algorithm optimization of datasets [32], due to its short sequencing reads; however, some errors, such as “false positives” [33], exist. The biological assessment of TCM preparation based on the NGS approach using both ITS2 and *trnL* biomarkers has been used to analyze Liuwei-Dihuang-Wan with an additional method as supplementary [34], but doubt regarding contamination existed and was not well explained. Since the lengths of some barcodes were out of the range of second-generation sequencing techniques, overlapping assembly was often used, from which algorithm errors might occur [26]. By contrast, long-read sequencing approaches, such as single-molecule real-time (SMRT) sequencing, which can output longer sequencing reads directly, have been shown to be competitive and also to exhibit more enrichment information and higher identification efficiency [35,36]. With the development of science and technology, they have emerged as potential approaches due to advantages of longer sequencing reads and deeper sequencing depth [37,38]. Long-read sequencing techniques have already been used in some aspects, such as microorganism identification [39,40] and taxonomic profiling [41], and have provided an economical way to monitor the legality and safety of traditional patent medicines [42,43]. Nevertheless, these studies have not yet elucidated this novel authentication approach for Chinese herbs in an adequate and well-controlled methodological analysis.

In the current study, basing on the TcmBarcode database and long-read sequencing technology, we put forward Full-length Multi-barcoding (FLMB), a method that capable of identifying the biological origins of multi-species mixture and could elucidate further details of DNA barcoding. This method was applied to the identification of *Sheng-Mai-San* (SMS), a classical prescription whose ingredients could be well controlled by hand-making, through SMRT sequencing of the amplicons of ITS2 (the second internal transcribed spacer of nuclear ribosomal DNA) and *psbA-trnH* (a chloroplast gene), two barcodes which have been recommended as standard DNA markers that are widely used in herbal medicine [44,45]. Moreover, *Sanwei-Jili-San* (SJS), another herbal compound that contains animal ingredient, has been submitted to be analyzed with CO1 (cytochrome c oxidase subunit 1, a mitochondrial gene) and ITS2 as a verifying approach.

## 2. Materials and Methods

### 2.1. Sample Collection and Powders Preparation

Raw materials of Ginseng Radix et Rhizoma (dried roots and rhizomes of *Panax ginseng* C. A. Mey., RS1/2/3), Ophiopogonis Radix (dried roots of *Ophiopogon japonicus* (L. f) Ker-Gawl.; MD1/2/3/4), Schisandrae Chinensis Fructus (dried fruits of *Schisandra chinensis* (Turcz.) Baill., WW1/2/3), Malvae Fructus (dried fruits of *Malva veriticillata* L., DK) and Tribuli Fructus (dried fruits of *Tribulus terrestris* L., JL), were purchased from production places, medicinal materials markets or companies; Fresh crab of *Eriocheir sinensis* H. Miline–Edwalds (FH), was purchased from a local supermarket (Table 1). All raw materials were identified by their morphologies, according to the Chinese Pharmacopeia [46]. Biological origins of these decoction slices were further tested by DNA barcoding through Sanger sequencing with barcodes of ITS2 and *psbA-trnH* for the flora, while CO1 for the fauna.

Preparation of the powder *Sheng-Mai-San* (SMS): decoction slices of RS1, MD3 and WW2 were mixed by weight with a ratio of 3:3:2 and then ground into powder (SMS1/2/3). Mixed powder (SJS1/2/3) of *Sanwei-Jili-San* (SJS) was manufactured by decoction slices of DK, JL and FH with a weight ratio of 3:5:3. The reducing and samples collection (around 5g for each powder sample) of all these powders were performed by quartering according to the general principle ‘0211’ in Chinese Pharmacopeia [46].

### 2.2. DNA Preparation and Sanger Sequencing

DNA of the powders (200 mg per sample) and independent ingredients (50 mg per sample) in Table 1 were extracted using the Plant Genomic DNA Kit (Tiangen Biotech Co., Ltd, Beijing, China), respectively. PCR systems containing 1 × *Taq* MasterMix (Aidlab Biotechnologies Co., Ltd., Beijing, China), 1 μM of each primer [47,48,49,50] (primer information see in Supplementary Materials Table S1) and ~100 ng DNA templates, were performed using conditions as followed: 95 °C for 4 min; 94 °C for 30 s, 55 °C for 1 min, 72 °C for 1 min, 35 cycles; and 72 °C for 10 min for ITS2 and *psbA-trnH* [8,12,50], while 94 °C for 1 min; 94 °C for 1 min, 45 °C for 1.5 min, 72 °C for 1.5 min, 5 cycles; and 94 °C for 1 min, 50 °C for 1.5 min, 72 °C for 1 min, 35 cycles; 72 °C for 5 min for CO1 [47]. Sanger sequencing of those PCR products were performed to confirm their biological origin. Then DNA mixtures in Table 1 of both formulas were prepared from the DNA samples of their ingredients, all of which were identified by DNA barcoding.

At the same time, four PCR products of SMS’ ITS2 fragment were purified using the MinElute^®^ Gel Extration Kit (Cat. No. 28606, Qiagen, Hilden, Germany). Then these purified fragments were inserted to the pMD 19-T vector (Takara, Beijing, China), transferred into competent cells of *E. coli* and selected through blue-white spot screening. Finally, a total of 81 white clones obtained were grown in liquid culture and then sequenced by Sanger method, as a comparison for SMRT sequencing.

### 2.3. Amplicon Libraries Preparation for SMRT Sequencing

**Figure 1:**
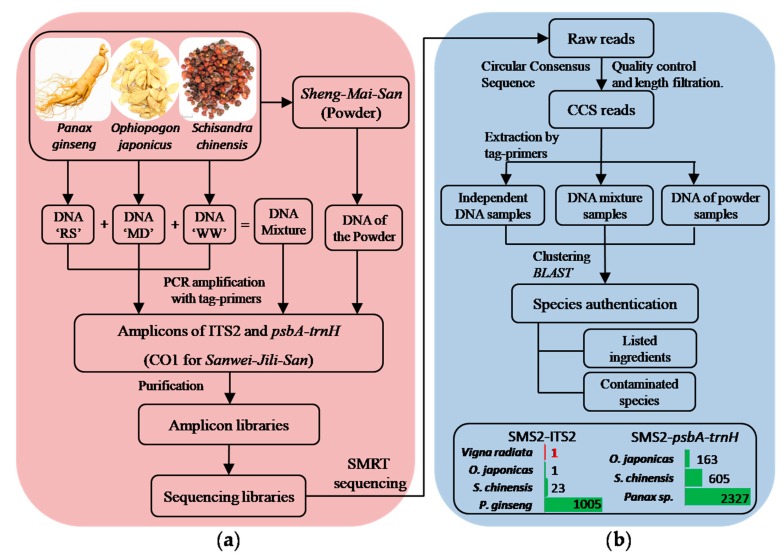
Pipeline of Full-length Multi-barcoding, analysis of SMS samples as an example. (**a**) sample preparation, (**b**) data analysis. (This figure was drawn by Peng Zhang).

As shown in Figure 1, DNA samples were prepared in three groups: (1) DNA extracted from raw materials; (2) mixtures of the DNA from raw materials, by a volume ratio of 3:3:2 (RS-MD-WW); (3) DNA extracted from the powders.

All DNA samples were used as templates for PCR amplification of ITS2 and *psbA-trnH*, respectively. Amplification for each amplicon were carried out using different pairs of tag-primers, to which several protective bases and labeling bases were attached to the 5′ end of the conventional primers (tag-primers’ sequences were shown in Supplementary Table S1, pairs of tag-primers corresponding to different amplicons were shown in Supplementary Materials Table S2). After electrophoresis, positive PCR products were purified using Agencourt^®^ AMPure^®^ XP beads (Beckman Coulter, Brea, CA, USA) by 0.8× volume and the concentrations of those purified amplicons was determined using a Qubit^®^ 3.0 Fluorometer (Invitrogen by Life Technologies, Carlsbad, CA, USA). In the end, purified fragment amplicons were pooled together to form sequencing libraries by certain quantity of nucleic acids, 500 ng for per fragment amplicons amplified from group 2 and 3, and 200 ng for those from group 1 (distribution of those amplicon libraries in sequencing libraries are shown in Table 1 and Supplementary Table S2).

### 2.4. SMRT Sequencing and Data Analysis

The sequencing libraries underwent chemical process with the SMRTbell^TM^ Template Prep Kit 1.0, and then were bound with V2 primers using the DNA/polymerase Binding Kit P6 V2 and P6- DNA polymerase, respectively. Next, each library was transferred to a 96-well PCR plate for real-time sequencing with C4 reagents on a PacBio II SMRT sequencing platform (Pacific Biosciences of California, Menlo Park, CA, USA).

Following the SMRT Analysis pipeline (v5.0.1), the resulting bas.h5 files were analyzed to generate Circular Consensus Sequence (CCS) passing reads. CCS parameters as follows: Minimum Full Passes = 7, Minimum Predicted Accuracy = 90, Minimum and Maximum Reads Length of Insert (In Bases) = 200 and 800). Resulting reads of each amplicon were extracted from CCS-pass reads according to corresponding tag-primer pairs. Along with the data analysis pipeline, data size, amount and length of the reads that remained available were carried out. Based on previous reports that error profile is about 13% for single pass in SMRT sequencing, the error profile of each locus in the resulting reads for 7-pass is estimated, in theory, to be around 0.72%

$$\overset{7}{\underset{k=4}{\sum }}C_{7}^{k}\times 0.13^{k}\times {\left(1-0.13\right)}^{7-k}$$

This will decline following an increase of the circular number. At the same time, the resulting reads in an amplicon that belonging to the same species were analyzed to define the profile and its influence on biological identification.

### 2.5. Clustering and Biological Identification of Resulting Reads

CodonCode Aligner (v5.1.5.3) was used to perform sequences alignment for resulting reads of each amplicon, with parameters set as followed: end to end alignments, Min. percent identity = 95.0, Min. overlap length = 200, Min. score = 150 (for CO1: Min. overlap length = 500, Min. score = 400). After verifying by labeling bases and trimming by tag-primers, the sequences of contigs and unassembled reads generated from assembly for each amplicon were submitted to perform BLAST (Basic Local Alignment Search Tool) against two public databases, the TcmBarcode system and the GenBank nucleotide Non-redundant database (https://www.ncbi.nlm.nih.gov/) [51], in which Max. score ≥ 400 and Identities ≥ 90% of the top hits for each sequence was defined as an effective one [52].

At the same time, four PCR products of SMS’ ITS2 fragment were purified using the MinElute^®^ Gel Extration Kit (Cat. No. 28606, Qiagen). Then, these purified fragments were inserted to the pMD 19-T vector (Takara), transferred into competent cells of *E. coli* and selected through blue-white spot screening. Finally, a total of 81 white clones obtained were grown in liquid culture and then sequenced by Sanger method. Species identification for these clones was performed, as comparative analysis with SMRT sequencing.

The definitions of the resulting reads, in contigs or unassembled ones achieving from each same amplicon but being assigned to different species, are as below.

- Original: A resulting read is original if it was assigned to the biological species of a sample.
- Endogenous: A resulting read is endogenous if the species which it was assigned to could be found in the raw materials, though it was not original.
- Exogenous: A resulting read is exogenous if it was assigned to other species which was considered as contamination from the outside, but not the biological species of the raw material or the powder.
- Invalid: A resulting read is invalid if it could not be assigned to biological species due to a lower similarity with the sequences in both databases, which was also defined as noneffective, rather than the effective ones that were defined as *original*, *endogenous* or *exogenous*.

### 2.6. Method Testifying by SJS

In order to test the feasibility of our method, *Sanwei-Jili-San* (SJS) was involved in our study. SJS is a Mongolian proved recipe that contains both herbal and animal ingredients, including Malvae Fructus (dried fruits of *Malva veriticillata* L., DK), Tribuli Fructus (dried fruits of *Tribulus terrestris* L., JL), Chinese fresh-water crab (*Eriocheir sinensis* H. Miline-Edwalds, FH). Its ingredients and their DNA mixture (JH) were comparatively analyzed using the barcodes of ITS2 and CO1, while the latter was added for the identification of animal ingredient.

## 3. Results

### 3.1. Species Authentication by Sanger Sequencing

ITS2 and *psbA-trnH* regions of SMS and relevant independent raw materials were successfully amplified. Sanger sequencing of these PCR products demonstrated that all the raw materials were from correct original species. Interestingly, consensus sequences of both regions of SMS from Sanger sequencing were the same with those of *Panax ginseng* (RS). The raw materials of SJS was similarly tested with ITS2 and CO1.

The acquisition of ITS2 fragments of *Ophiopogon japonicus* (MD) was difficult but not impossible, due to its unsatisfactory amplifying efficiency. And 10 PCR cycles as well as two more parallel tests were individually added as a compensation to achieve sufficient concentration for SMRT sequencing. In total, five amplicon libraries of MD’s ITS2 were prepared and sequenced, four of which the PCR cycles were 35 + 10.

### 3.2. Analysis Results Via FLMB

#### 3.2.1. Data Processing of SMRT Sequencing

A total of three sequencing libraries were sequenced by SMRT sequencing (Flow cell A, B and C for Library A, B and C respectively). For Library A as an example, which contained 12 purified amplicons of SMS and HSM, 57,147 raw reads were yield up with an average length of 31,217 bp. After CCS processing, 36,497 CCS-pass reads were produced, among which 25,877 resulting reads were extracted to their belonging amplicons according to corresponding sequences of tag-primer pairs. Reserving rate was about 45.3% (25,877/57,147). The datasets used during the current study are available via NCBI under the project number PRJNA419289 (SUB3240739). Details for the data processing information of Library A are shown in Table 2.

#### 3.2.2. Species Identification of SMS by SMRT Sequencing

**Figure 2:**
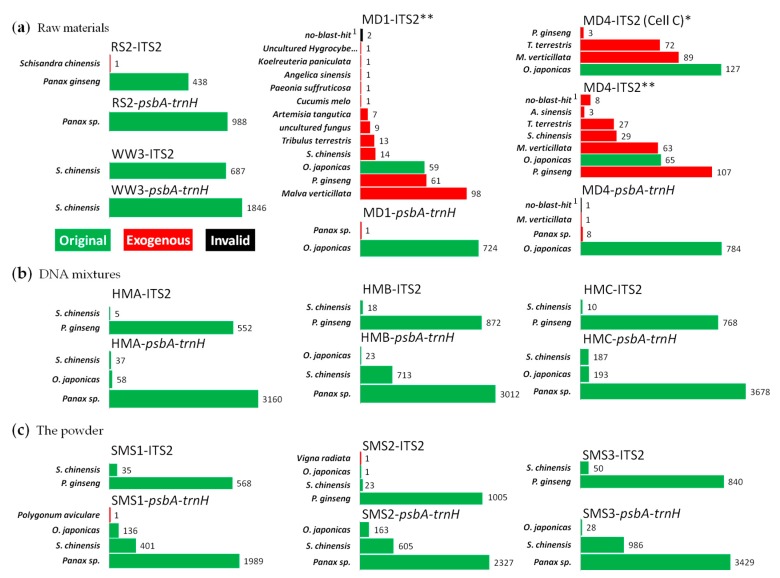
Analysis results on *Sheng-Mai-San* by full-length multi-barcoding. (**a**) Representative results of the raw materials; (**b**,**c**), results of the HSM and SMS, respectively. **, PCR cycles were 35 + 10; *, PCR cycles were 35. *Panax sp.*, reads that cannot BLAST to certain species in genus of Panax; *no-blast-hit* ^1^, resulting reads that with Max. score < 400 or Identities < 90% for top hits in BLAST.

The resulting reads for each amplicon were clustered and aligned by CodonCode Aligner, and amplicon-corresponding labels were used to validate the correct belonging of these resulting reads. Then the species identifications were carried out through BLAST function in TcmBarcode System and NCBI. Species with nearest match and the number of resulting reads for each amplicon are shown in Figure 2.

Species identification results of the reads from raw materials were as expected (Figure 2a), except for the ITS2 amplicons of MD, in which several exogenous reads were present (all results were shown in Supplementary Table S3). However, almost no exogenous reads were detected in their *psbA-trnH* amplicons and, in both ITS2 and *psbA-trnH* amplicons of the HSM samples, which were all prepared from each same DNA samples of MD as a raw material. No exogenous species were detected (Figure 2b). So, these contaminations in MD’s ITS2 amplicons were considered to be introduced during PCR amplification, thus an additional 10 PCR cycles were likely to bring in more exogenous reads or exogenous species.

For multi-species mixtures, ITS2 amplicons of SMS and HSM showed resulting reads identified as *Panax ginseng* and *Schisandra chinensis*, while *psbA-trnH* showed *Panax sp.*, *S. chinensis*, and *Ophiopogon japonicus*, in summary (Figure 2b,c). For example, in ITS2 amplicons of SMS3, there were 840 and 50 resulting reads for *P. ginseng* and *S. chinensis*, while in *psbA-trnH* amplicons of SMS3, there were 3429, 986 and 28 resulting reads for *Panax sp.*, *S. chinensis* and *O. japonicus*, respectively. The ITS2 sequence of *O. japonicus* nearly could not be found in results of all these mixtures, while the *psbA-trnH* sequence of *P. ginseng* could hardly be distinguished from those of some closely related species such as *P. japonicus* or *P. quinquefolium* (American ginseng). 

On the other hand, amplicons of DNA mixtures (HSM) were extremely pure; therefore, no exogenous species were detected, while exogenous sequences such as *Vigna radiata* and *Polygonum aviculare* were found in those of SMS, whose ingredients were in higher cleanliness level after processing.

#### 3.2.3. Result of Clone-Dependent Sanger Sequencing

**Figure 3:**
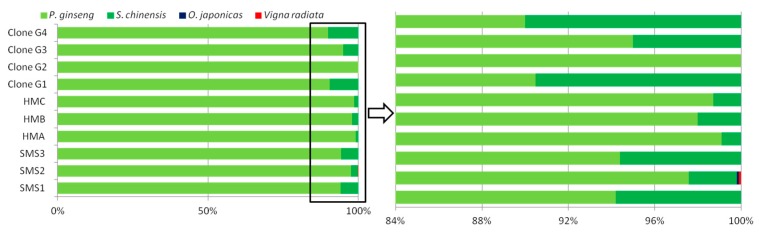
Percentage of different raw materials’ reads in ITS2 (the second internal transcribed spacer of nuclear ribosomal DNA) amplicons of SMS. HMA/B/C and SMS1/2/3 were analyzed by FLMB, while Clone G1/G2/G3/G4 were analyzed by clone-dependent Sanger sequencing.

A total of 81 clones from four PCR products of SMS’ ITS2 region were picked out for Sanger sequencing. Among them, 76 clones were identified as *P. ginseng*, and 5 as *S. chinensis*. Yet, no clone belonging to *O. japonicus* was detected in these selected clones, which was similar with the results of SMRT sequencing (Figure 3). Clone-dependent Sanger sequencing showed some randomness due to its low throughput that even 20 clones, such as Clone G2 (Figure 3), could not make a confident checkout of *S. chinensis* (its abundance in ITS2 amplicons of SMS was estimated to around 4.3% via the analysis of SMRT sequencing). SMRT sequencing allowed us to obtain ITS2 fragment of *O. japonicus*, in spite of its negligible abundance (below 0.1%).

#### 3.2.4. Results for SJS

**Figure 4:**
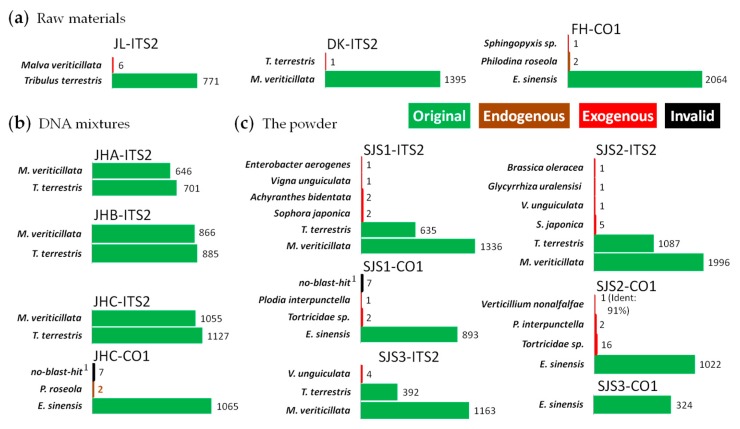
Analysis results on *Sanwei-Jili-San* by full-length multi-barcoding.

To further test the feasibility of this method, we performed our approach on *Sanwei-Jili-San* with barcodes of ITS2 and CO1. In summary, all the raw materials in the powder and their DNA mixtures could be successfully identified at species level (Figure 4). In the CO1 amplicons of the *Eriocheir sinensis* (FH) and JHC (DNA mixture), several reads were identified as *Philodina roseola* [53], which is often used in crab breeding and is considered as an endogenous species. In contrast, in a few reads from exogenous animal species, such as *Plodia interpunctella* [54], an insect commonly seen in storage spots, and *Tortricidae sp.* [55,56], a class of harmful insects in agriculture field, was found in the powders.

A total of nine kinds of exogenous biological sources, including five plant species, two animal species and two microorganism species, were found in the powders of *Sanwei-Jili-San* (SJS) by the combination of CO1 and ITS2 (Figure 4c). But no exogenous species were detected in these amplicons of its DNA mixtures (Figure 4b), which was similar with the results of HSM, leading to the conclusions that these contaminations were indeed existing in the powder. In conclusion, FLMB is an effective analysis method for biological mixtures.

## 4. Discussion

### 4.1. Methodological Analysis

Procedures’ quality control, optimized data-processing parameters, and other methodological analysis in these adequate and well controlled studies, all had provided more details on DNA barcoding that could contributed to a better understanding. And some more abundant but precise information could be carried out at the same time, via full-length multi-barcoding, a feasible, effective and accuracy method.

#### 4.1.1. Precise Definitions from High Accuracy Resulting Reads

A relatively lower sequencing accuracy for single pass (about 13% error rate) [37] had once hindered the application of SMRT sequencing. But as the fragment lengths of frequently-used DNA barcodes were short (about 200 to 1500 bp), long-read sequencing strategy enables self-correction by CCS as the read length of SMRT sequencing could reach 20 kb in average [37], and thus, the sequencing accuracy could be improved [57]. In fact, the error properties of Pacific Biosciences sequencing technology was defined as free of the context-specific effects, which may affect other sequencing technologies [58], and it has already shown excellent utility in some aspects such as SNP discovery [59].

**Figure 5:**
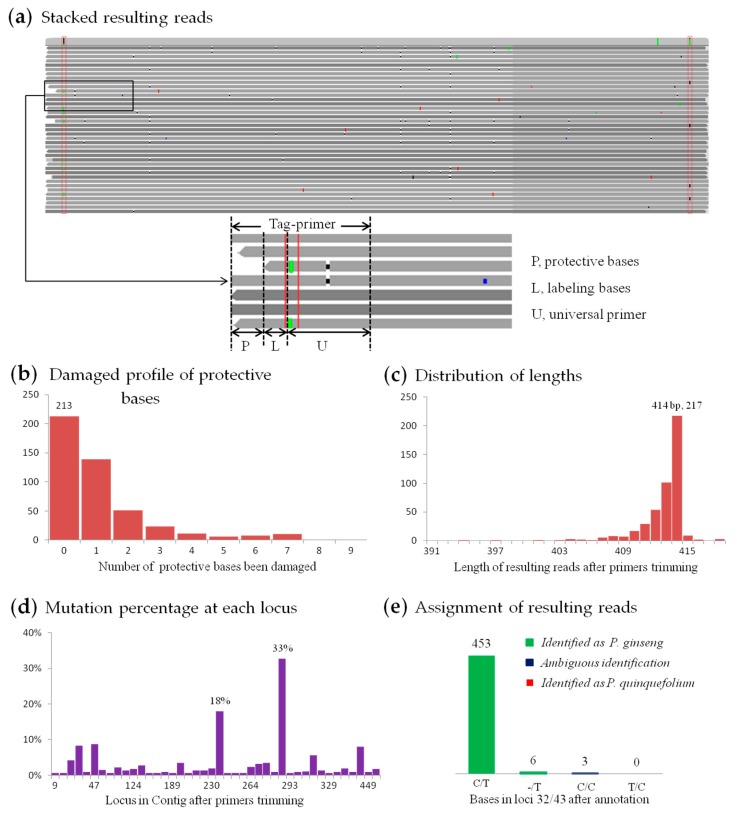
Analysis of 462 resulting reads from the ITS2 (the second internal transcribed spacer of nuclear ribosomal DNA) amplicon of RS3. (**a**) Part of stacked resulting reads; red boxes mark degenerate bases in tag-primers whose constitution shows in the smaller block below. (**b**) Damaged profile of protective bases, as the bases missing at both ends in (**a**). (**c**) Length distribution after primers trimming. (**d**) Mutation percentage at each loci (only shows those with percentage higher than 0.5%). (**e**) Species assignment results.

In this study, 462 resulting reads were screened out for the ITS2 amplicon from RS3. And a total of 812 bases (0.39%) with ‘mutations’ at 208 positions were found in 210,210 bases after primer trimming (Figure 5a), which should involve all ‘mutations’ from multi-copy gene, amplification mismatches and sequencing error. Therefore, the error profile was actually below 0.39%, and was significantly lower than the expectation (0.72% for 7 CCS passes), because the mean number of CCS passes had reached 40. Mutation percentage for each position shows in Figure 5d, which can be used to find homologous sequence reads from closely related species. Although there is a close taxonomy relationship between *Panax ginseng* and *P. quinquefolium*–only two different loci in their ITS2 regions [60], there were no resulting reads that might be assigned to *P. quinquefolium* (Figure 5e), reflecting a high accuracy rate of this method. And since there was a steady different site between the *psbA-trnH* sequences of “Zhe” *Ophiopogon japonicus* and “Chuan” *O. japonicus*—two different variance-types, genotype in resulting reads from different amplicons were verified (Supplementary Table S4). As a conclusion, high accuracy resulting reads generated by SMRT sequencing and CCS could lead to precise and reliable identification results.

#### 4.1.2. Every Resulting Reads Counts

By using a combination of proper DNA barcodes, all the biological origins of raw materials in *Sheng-Mai-San* and *Sanwei-Jili-San* were validated with accurate identification, while some exogenous or endogenous species were detected at the same time. For all the 46 amplicons sequenced, only 7 of them had *no-blast-hit* (top hits with low Max. score or low Identities) resulting reads, that 85% (39/46) achieved resulting reads with 100% effective rate, which means all the resulting reads could be explained. And the effective rate of all resulting reads from the 46 amplicons was 99.93% (60,191/60,232), i.e., almost every resulting read was counted (Table 3). Although still unknown, some of these *no-blast-hit* resulting reads were believed to exist, but had not yet been submitted to both databases used in this study, because most of them distributed in amplicons of MD, while a few were homologous.

#### 4.1.3. Recommended Sequencing Depth

**Figure 6:**
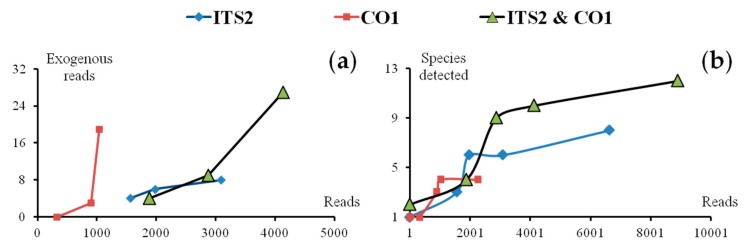
Number of exogenous reads and exogenous species rise along with the increasing of sequencing depth. (**a**) Relations between exogenous reads and total resulting reads. (**b**) Relations between species detected and total resulting reads. ITS2: the second internal transcribed spacer of nuclear ribosomal DNA), CO1: cytochrome c oxidase subunit 1.

Raw materials of the SMS were well cleaned before powdering. However, raw materials of *Sanwei-Jili-San* (SJS), like JL, were hard to clean in mass production because of their tiny size, i.e., lots of exogenous reads were found as a consequence. The quantitative relationship between the exogenous reads, the species detected with the total resulting reads were analyzed, i.e., more exogenous reads—or even more species before reaching a ceiling—were likely to be detected as the number of total resulting reads increasing (Figure 6). In general, 1000 resulting reads were recommended as a proper sequencing depth for each amplicon library of formula; thus, a single SMRT sequencing cell covered more than 20 amplicon libraries, and the cost per multi-species mixture rapidly decreased.

#### 4.1.4. Combination Makers Makes Identification More Accuracy and Entirely

Markers combination could not only enhance the discrimination ability, but could also promote the possibility of successful amplification for templates from different biological original materials [61,62]. The *psbA-trnH* resulting reads of *P. ginseng* could hardly be distinguished from those of other *Panax* spp., while ITS2 reads showed the only biological origin, *P. ginseng*, which indicated the importance of appropriate regions for DNA barcoding. ITS2 sequences of *O. japonicus* could hardly be detected in amplicons of SMS or HSM due to its unsatisfactory PCR efficiency, while the *psbA-trnH* could be successfully amplified and detected, despite its different variance-types (Table S2). Moreover, a combination of CO1 and ITS2 revealed more species for the samples of *Sanwei-Jili-San* (SJS), not merely endogenous and exogenous, but also covering a vast number of species, ranging from plant, animal to microbial. For Chinese herbal formula that are preparation of plant, animal, fungi and minerals medicine, combination of DNA barcodes can facilitate more accurate and integrated authentication of biological origins.

#### 4.1.5. Quality Control of the Procedures

Compared with a low effective rate of raw reads (~20.3%) in a former study [42], the effective rate of raw reads in this study was significantly enhanced to a much higher level (~45.3%), partly due to the protective bases designed in this study. And as suspect unauthentic contaminations or negative results of some species occurred in other studies [9,34], further research with adequate and well-controlled studies were needed. The method proposed in this study had provided technical support for the study on Danggui Buxue Formula [52], while the latter formed an overall quality evaluation system for herbal medicine, including subsequent quantitative analysis through high performance liquid chromatography.

To ensure the reliability of the identification results, several measurements for quality control of the library preparation and data analysis were taken. (1) All raw ingredients were purged before DNA extraction and their biological origins of herbs were confirmed by Sanger sequencing. (2) Analyses of the powders of SMS and SJS via FLMB were carried out by adequate and well-controlled studies, i.e., three batches for each formula were used, compared with a collateral test of the raw materials and their DNA mixture. (3) Tag-primers were designed, which contained labeling bases that were used to recognize which sample a particular read was derived from, and protective bases that were used to protect these labeling bases with which they joined due to damage. (4) Gel electrophoresis, beads purification and concentration determination were all used to achieve purified fragments with enough concentration. (5) After read extractions, all the resulting reads were examined by tag-primers to guarantee their correct ownership; the parameters of CCS, the damaged profile of protective bases and the error rate of resulting reads were all analyzed. (6) Two databases, TcmBarcode System, a professional identification system for herbs, and the GenBank sequence database which contains tremendous DNA sequences ranging from prokaryotes to eukaryotes, were simultaneously used to obtain creditable results.

### 4.2. Different Sequencing Technologies Applied to DNA Barcoding

**Figure 7:**
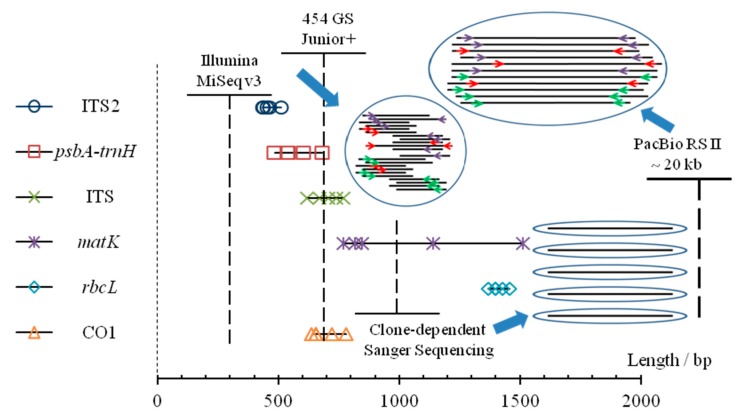
Comparative analysis of different sequencing platforms applied to DNA barcoding. Lengths of amplicon fragments of five barcodes regions (ITS2, *psbA-trnH*, ITS, *matK*, *rbcL*) for five vegetable materials in two studied formulas, and lengths of CO1 for five animal materials (including crabs) have been shown as marks. For each sequencing platform, the dotted vertical line represents the read length while the solid transverse line represents the sequencing throughput. Though the number of raw reads obtained from the platform of PacBio is less than those of the other two NGS platforms, its advantages of long-read sequencing could maintain more original and effective information.

As shown in Figure 7, Sanger sequencing, though low in throughput but providing relatively long and accurate reads, has been widely used in authentication for single species sample [63]. As for complex biotic mixtures, high-throughput sequencing has eclipsed clone-dependent Sanger sequencing over the past decade [36]. For NGS with short read sequencing, identification results were suspicious sometimes, when problems emerged when overlapping was used [26]. This method, FLMB, is a perfect interpretation for DNA barcoding through adequate and well-controlled experiments (Table 4), that could achieve complete and accurate identification for biotic components [42,52], and it could shed some light on the negative or false positive results in some studies of DNA barcoding.

Long reads obtained from the SMRT sequencing could simplify the subsequent data analysis process by sequencing through the whole fragment of DNA barcodes repeatedly. Thus, the efficient rate of the resulting reads from SMRT sequencing could reach 100%, and a more reliable conclusion can be drawn in the end. Furthermore, the tag-primers designed in this study contain both labeling bases (6 bp) and protective bases (8 bp). The former ones, located in the middle of tag-primers, were group-specific, corresponding to different sample. The latter, at the 5′ end and all the same for different tag-primers in this study, could protect the labeling bases from damage by taking its outing place and maintain a high effective rate of the raw reads, that was similar but not the same with the sequences used to protect DNA from exonucleolytic degradation [64]. Besides, this strategy of multiplexing allows sequencing multiple samples simultaneously and guarantees a high-level retention rate from raw reads to resulting reads in the process of data manipulation, thus help to reduce the sequencing cost. On the contrary, the cost of Sanger sequencing for 20 clones is equivalent to one eighth the cost of a SMRT sequencing cell which may contain tens of amplicon libraries, not to mention the cumbersome and time-consuming process of cloning which was unlikely to find rare fragments [65].

### 4.3. Applications and Challenges

A comprehensive identification of biological origin for herbs is absolutely necessary because most herbal products are multi-species mixtures, such as the herbal compound formula and those being labeled as single ingredient but containing fillers or substitutions [9,66]. As shown in the results of SMS, the gene copy number from different species and PCR bias for different fragments was of varying significance, which might lead to the detection of those species with more gene copies or higher PCR efficiency. In contrast, parameters in the pipeline of DNA barcoding, especially barcode selections and PCR conditions, should be taken into account because they could make some difference to the final results, especially when samples tested were multi-species mixtures. Based on biological composition information as a qualitative analysis, a quantitative analysis could be carried out through other approaches such as chemical analysis. Thus, a more integrated quality evaluation system [4,52] could be established, from which a more accurate but credible identification result, including an objective definition of contamination, substitution and adulteration, will be obtained.

From these comparative analyses of the formula samples, the raw materials and their DNA mixtures by FLMB, a perfect interpretation on DNA barcoding for both single-species samples and multi-species samples was achieved, which will promote DNA barcoding from single-species ingredient to multi-species mixtures. As it can be used to detect most species of biotic components, a better assessment of Chinese medicine, not only for its safety clinical use but also for the conservation of protected species [67,68] could be carried out. What’s more, as some microbial species were also detected. This method could even be used to find possible microorganism that have potential toxicity, such as *Aspergillus flavus* and *A. parasiticus* fungi which may produce aflatoxins [69], by using proper barcodes. It could also provide a novel insight into the biodiversity analysis on other research areas [70]. While multigene was an effective way to promote the successfully identifying rate [71] for single-species, multi-barcoding could be used to find more species with more precisely identification for multi-species mixtures and in this study, a recommended sequencing depth was put forward.

In the current study, two powerful reference databases were employed to guarantee the reliability of identifying results which was considered as a challenge a few years ago [9], while another challenge exits, namely, the detection of the ingredients whose DNA was degraded, as parts of Chinese patent medicine covers procedures that may lead to DNA’s damage such as heating. Other than some species-specific methods [34,72], FLMB using short segments, though they might not have an equally distinguishing ability with the longer ones, as barcodes, should be taken into consideration for their potentially superior amplification.

## 5. Conclusions

A considerable number of classical preparations in TCM are still widely used [73], such as SMS and SJS [74,75]. Unlike raw materials and decoction slices whose biological identification is relatively simple because DNA barcoding and other approaches may easily be employed, biological identification of samples which contain multi-species ingredients is more challenging, and almost no methods have been adopted to address at this issue in Chinese Pharmacopeia. But the substitution and adulteration in herbs and other supplements [76], whether intentionally or unintentionally, leaves nonnegligible health risks for consumers, such as known organisms that have known toxicity, side effects, allergens and/or negatively interact with other herbs, supplements, or medications [9].

In this study, we established a novel method, Full-Length Multi-Barcoding (FLMB), for biological analyses of multi-species mixtures. The proposed methodology was carried out with adequate and well-controlled studies, by analyzing a classic formula *Sheng-Mai-San* with barcodes of ITS2 and *psbA-trnH*, comparing with its raw materials and their DNA mixtures. Extremely pure amplicons for both the single-species and the multi-species were successfully achieved, yielding more more scientific and believable results by DNA barcoding. The results of another formula, *Sanwei-Jili-San* using ITS2 and CO1, showed that this method was feasible and reproducible. In conclusion, the method could provide a powerful and credible approach for the biological analysis of complex biotical mixtures, covering a vast number species ranging from plants, to animals to microbes.

## Figures and Tables

**Table 1:** Preparation of the DNA samples and their information.

Formula	Samples (and Its Biological Origin)	DNA ID	Notes
*Sheng-Mai-San*	Ginseng Radix et Rhizoma (*Panax ginseng*)	RS1	Company A
		RS2	Company A
		RS3	Company A
	Ophiopogonis Radix (*Ophiopogon japonicus*)	MD1	‘Zhe’ *O. japonicus*; Cixi, Zhejiang province; producing area
		MD2	‘Zhe’ *O. japonicus*; Xiangshan, Zhejiang province; wildness
		MD3	‘Chuan’ *O. japonicus*; Santai, Sichuan province; Market B
		MD4	‘Chuan’ *O. japonicus*; Sichuan province; Company C
	Schisandrae Chinensis Fructus (*Schisandra chinensis*)	WW1	Company C
		WW2	Company C
		WW3	Company C
	*Sheng-Mai-San* (SMS)	SMS1	Mixed powder
		SMS2	Mixed powder
		SMS3	Mixed powder
	DNA mixture (HSM)	HMA	DNA Volume of MD1-RS1-WW1 = 3:3:2
		HMB	DNA Volume of MD2-RS2-WW2 = 3:3:2
		HMC	DNA Volume of MD3-RS3-WW3 = 3:3:2
*Sanwei-Jili-San*	Malvae Fructus (*Malva veriticillata*)	DK	Company D
	Tribuli Fructus (*Tribulus terrestris*)	JL	Company D
	Chinese fresh-water crab (*Eriocheir sinensis*)	FH	A local supermarket
	*Sanwei-Jili-San* (SJS)	SJS1	Mixed powder
		SJS2	Mixed powder
		SJS3	Mixed powder
	DNA mixture (JH)	JHA	DNA Volume of DK-JL-FH = 3:5:3
		JHB	DNA Volume of DK-JL-FH = 3:5:3
		JHC	DNA Volume of DK-JL-FH = 3:5:3

**Table 2:** Processing information for library A’s data generated by SMRT sequencing.

	Raw Data	Circular Consensus Sequence Filtration	Extraction
Reads	57,147	36,497	25,877
Total bases	1.784 × 10^9^	19,308,858	14,117,133
Data size	24.4 GB	21.3 MB	13.7 MB
Mean length	31,217 bp	529 bp	546 bp
Length range	0~70 kb	200~799 bp	348~799 bp

**Table 3:** Amplicons’ information in different SMRT cells and effective rate for each amplicon.

SMRT Cell	DNA ID	Pair of Tag-Primers ^#^	Resulting Reads	*No-Blast-Hit*^1^ Resulting Reads	Effective Rate
Cell A	HM1	T17	3255	0	100.00%
	HM2	T18	3748	0	100.00%
	HM3	T19	4058	0	100.00%
	SMS1	T13	2527	0	100.00%
	SMS2	T14	3095	0	100.00%
	SMS3	T15	4443	0	100.00%
	SMS1	i13	603	0	100.00%
	SMS2	i14	1030	0	100.00%
	SMS3	i15	890	0	100.00%
	HMA	i17	557	0	100.00%
	HMB	i18	890	0	100.00%
	HMC	i19	778	0	100.00%
Cell B	RS1	i5	279	0	100.00%
	RS2	i6	439	0	100.00%
	RS3	i7	462	0	100.00%
	WW1	i9	431	0	100.00%
	WW2	i10	706	0	100.00%
	WW3	i11	686	0	100.00%
	MD1	T1	725	0	100.00%
	MD2	T2	578	0	100.00%
	MD3	T3	749	0	100.00%
	MD4	T4	794	1	99.87%
	MD1 **	i1	268	2	99.25%
	MD2 **	i2	512	7	98.63%
	MD3 **	i3	568	14	97.54%
	MD4 **	i4	302	8	97.35%
	WW1	T9	1276	0	100.00%
	WW2	T10	445	0	100.00%
	WW3	T11	1846	0	100.00%
	RS1	T5	911	0	100.00%
	RS2	T6	988	0	100.00%
	RS3	T7	1617	0	100.00%
Cell C	MD3 *	i3	291	0	100.00%
	JL	i39	777	0	100.00%
	DK	i40	1396	0	100.00%
	FH	C9	2067	0	100.00%
	SJS1	i23	1977	0	100.00%
	SJS2	i22	3091	0	100.00%
	SJS3	i21	1559	0	100.00%
	SJS1	C1	898	2	99.78%
	SJS2	C2	1041	0	100.00%
	SJS3	C3	324	0	100.00%
	JHC	C6	1074	7	99.35%
	JHC	i32	2182	0	100.00%
	JHB	i31	1751	0	100.00%
	JHA	i24	1348	0	100.00%
Total			60,232	41	99.93%

Note: *no-blast-hit* ^1^, top hits with low Max. score (≤400) or low Identities (≤90%). **, PCR cycles were 35 + 10; *, PCR cycles were 35. ^#^,‘T’ as *psbA-trnH*, ‘i’ as ITS2 (the second internal transcribed spacer of nuclear ribosomal DNA), ‘C’ as CO1 (cytochrome c oxidase subunit 1).

**Table 4:** Cases of different sequencing approaches applied to DNA barcoding of herbs.

	Briefing	Reference
Sanger sequencing	The *trnH-psbA* could distinguish 18 species of Polygonaceae and their adulterants including 10 species that recorded in Chinese pharmacopoeia.	[10]
	The discrimination ability of ITS2, a most suitable region for DNA barcoding, were tested by more than 6600 plant samples and its successful identification rate was 92.7% at the species level.	[8]
	Most (26/44) of the North American herbal products tested contained DNA barcodes from plant species not listed on the labels. Sequences of different species had yield up from the same sample product.	[9]
	DNA barcoding was used to authenticate the components of antler powder in the market, while a few samples containing multi-species were analyzed by cloning method.	[14]
Short-read NGS	Amplicons of *trnL* and 16S from 15 TCM samples were sequenced in platform of Roche GS Junior, and over 49,000 amplicon sequence reads were generated. Many *trnL* sequence reads could not been precisely identified (most were just assigned to genera level), due to the limitation of read-length and reference database.	[17]
	HTS data for 30 *Liuwei-Dihuang-Wan* samples were generated based on 454 GS FLX Titanium sequencing. On averages, 3 and 2.4 prescribed species could be detected from a sample based on ITS2 and *trnL*, respectively. *Vigna* genus, a possible contaminated species, was detected in all three reference samples.	[34]
Long-read NGS	A total of 3703 and 4810 CCS reads from two reference and three commercial *Yimu-Wan* samples were mapped to the ITS2 and *psbA-trnH* regions, respectively. SMRT sequencing provides an affordable way to monitor the legality and safety of traditional patent medicines.	[42]
	A comprehensive quality evaluation system for herbal medicine was established, by combining two genetic-based approaches—third-generation sequencing and denaturing gradient gel electrophoresis, with analytical chemistry approaches.	[52]
	SMS and SJS have been analyzed through FLMB by an adequate and well-controlled methodological analysis, which shows perfect interpretation for DNA barcoding.	This study

## Supplementary Materials

The following are available online at https://www.mdpi.com/2073-4425/10/5/343/s1, Table S1: Information of primers and tag-primers, Table S2: Distribution of amplicons in different sequencing Cells and their corresponding tag-primers pairs, Table S3: Species identifying results on *Ophiopogon japonicus* by FLMB, Table S4: Validation of different locus between two types of *Ophiopogon japonicus* in *psbA-trnH* region.
