# Supplementary material for: Full-Length Multi-Barcoding: DNA Barcoding from Single Ingredient to Complex Mixtures
Source: Genes (Basel). 2019 May 7;10(5):343. doi: 10.3390/genes10050343 (PMC6562688; doi:10.3390/genes10050343)
Supplement: Supplementary file 1 [file genes-10-00343-s001.zip › Table S1. Information of universal primers and tag-primers.pdf]

Table S1. Information of universal primers and tag-primers

(**a**) Universal Primers

Barcode region	Primer	Sequence (5'-3')	Reference *
ITS2	P3 (Forward)	YGACTCTCGGCAACGGATA	(Cheng et al., 2016)
	E4 (Reverse)	RGTTTCTTTTCCTCCGCTTA	(Cheng et al., 2016)
psbA-trnH	PA (Forward)	GTTATGCATGAACGTAATGCTC	(Sang, Crawford, & Stuessy, 1997)
	TH (Reverse)	CGCGCATGGTGGATTCACAATCC	(Tate & Simpson, 2003)
CO1	LC (Forward)	GGTCAACAAATCATAAAGATATTGG	(Folmer, Black, Hoeh, Lutz, & Vrijenhoek, 1994)
	HC (Reverse)	TAAACTTCAGGGTGACCAAAAAATCA	(Folmer et al., 1994)

Note: * Cheng, Tao, Xu, Chao, Lei, Li, Li, Changhao, Zhang, Yu, & Zhou, Shiliang. (2016). Barcoding the kingdom Plantae: new PCR primers forITSregions of plants with improved universality and specificity. *Molecular Ecology Resources, 16*(1), 138-149. doi: 10.1111/1755-0998.12438; Folmer, O., Black, M., Hoeh, W., Lutz, R., & Vrijenhoek, R. (1994). DNA primers for amplification of mitochondrial cytochrome c oxidase subunit I from diverse metazoan invertebrates. *Molecular marine biology and biotechnology, 3*(5), 294-299. ; Sang, T., Crawford, D., & Stuessy, T. (1997). Chloroplast DNA phylogeny, reticulate evolution, and biogeography of Paeonia (Paeoniaceae). *Am J Bot, 84*(8), 1120. ; Tate, Jennifer, & Simpson, Beryl. (2003). *Paraphyly of Tarasa (Malvaceae) and Diverse Origins of the Polyploid Species* (Vol. 28).

(**b**) Tag-primers

Barcode region	Tag-primer	Sequence (5'-3')
ITS2	P3-1	ATCATCAGTCGACAYGACTCTCGGCAACGGATA
	P3-2	ATCATCAGTATCACYGACTCTCGGCAACGGATA
	P3-3	ATCATCAGTGCGTAYGACTCTCGGCAACGGATA
	P3-4	ATCATCAGACGCTAYGACTCTCGGCAACGGATA
	P3-5	ATCATCAGAGCTAGYGACTCTCGGCAACGGATA
	E4-A	ATCATCAGTCGACARGTTTCTTTTCCTCCGCTTA
	E4-B	ATCATCAGTATCACRGTTTCTTTTCCTCCGCTTA
	E4-C	ATCATCAGTGCGTARGTTTCTTTTCCTCCGCTTA
	E4-D	ATCATCAGACGCTARGTTTCTTTTCCTCCGCTTA
psbA-trnH	PA-1	ATCATCAGTCGACAGTTATGCATGAACGTAATGCTC
	PA-2	ATCATCAGTATCACGTTATGCATGAACGTAATGCTC
	PA-3	ATCATCAGTGCGTAGTTATGCATGAACGTAATGCTC
	PA-4	ATCATCAGACGCTAGTTATGCATGAACGTAATGCTC
	PA-5	ATCATCAGAGCTAGGTTATGCATGAACGTAATGCTC
	TH-A	ATCATCAGTCGACACGCGCATGGTGGATTCACAATCC
	TH-B	ATCATCAGTATCACCGCGCATGGTGGATTCACAATCC
	TH-C	ATCATCAGTGCGTACGCGCATGGTGGATTCACAATCC
	TH-D	ATCATCAGACGCTACGCGCATGGTGGATTCACAATCC

CO1	LC-1	ATCATCAGTCGACAGGTCAACAAATCATAAAGATATTGG
	LC-2	ATCATCAGTATCACGGTCAACAAATCATAAAGATATTGG
	LC-3	ATCATCAGTGCGTAGGTCAACAAATCATAAAGATATTGG
	LC-4	ATCATCAGACGCTAGGTCAACAAATCATAAAGATATTGG
	LC-5	ATCATCAGAGCTAGGGTCAACAAATCATAAAGATATTGG
	HC-A	ATCATCAGTCGACATAAACTTCAGGGTGACCAAAAAATCA
	HC-B	ATCATCAGTATCACTAAACTTCAGGGTGACCAAAAAATCA
	HC-C	ATCATCAGTGCGTATAAACTTCAGGGTGACCAAAAAATCA
	HC-D	ATCATCAGACGCTATAAACTTCAGGGTGACCAAAAAATCA
