# Supplementary material for: Full-Length Multi-Barcoding: DNA Barcoding from Single Ingredient to Complex Mixtures
Source: Genes (Basel). 2019 May 7;10(5):343. doi: 10.3390/genes10050343 (PMC6562688; doi:10.3390/genes10050343)
Supplement: Supplementary file 1 [file genes-10-00343-s001.zip › Table S2. Distribution of amplicons in different sequencing Cells and their corresponding tag-primers pairs.pdf]

Table S2. Distribution of amplicons in different sequencing Cells and their corresponding tag-primers pairs

SMRT Cell	DNA sample	Pairs code#	Tag-primer	
			Forward	Reverse
Cell A	HMA	T17	PA-5	TH-A
	HMB	T18	PA-5	TH-B
	HMC	T19	PA-5	TH-C
	SMS1	T13	PA-4	TH-A
	SMS2	T14	PA-4	TH-B
	SMS3	T15	PA-4	TH-C
	SMS1	i13	P3-4	E4-A
	SMS2	i14	P3-4	E4-B
	SMS3	i15	P3-4	E4-C
	HMA	i17	P3-5	E4-A
	HMB	i18	P3-5	E4-B
	HMC	i19	P3-5	E4-C
Cell B	RS1	i5	P3-2	E4-A
	RS2	i6	P3-2	E4-B
	RS3	i7	P3-2	E4-C
	WW1	i9	P3-3	E4-A
	WW2	i10	P3-3	E4-B
	WW3	i11	P3-3	E4-C
	MD1	T1	PA-1	TH-A
	MD2	T2	PA-1	TH-B
	MD3	T3	PA-1	TH-C
	MD4	T4	PA-1	TH-D
	MD1	i1	P3-1	E4-A
	MD2	i2	P3-1	E4-B
	MD3**	i3	P3-1	E4-C
	MD4	i4	P3-1	E4-D
	WW1	T9	PA-3	TH-A
	WW2	T10	PA-3	TH-B
	WW3	T11	PA-3	TH-C
	RS1	T5	PA-2	TH-A
	RS2	T6	PA-2	TH-B
	RS3	T7	PA-2	TH-C
Cell C	MD3*	i3	P3-1	E4-C
	JL	i39	P3-10	E4-C
	DK	i40	P3-10	E4-D
	FH	C9	LC-3	HC-C
	SJS1 (*Sanwei-Jili-San*)	i23	P3-6	E4-C
	SJS2	i22	P3-6	E4-B
	SJS3	i21	P3-6	E4-A
	SJS1	C1	LC-1	HC-A
	SJS2	C2	LC-1	HC-B
	SJS3	C3	LC-1	HC-C
	JHC (DNA mixture)	C6	LC-2	HC-C
	JHC	i32	P3-8	E4-D
	JHB	i31	P3-8	E4-C
	JHA	i24	P3-6	E4-D

Note: #, 'T'-*psbA-trnH*; 'i'-ITS2; 'C'-CO1. */**, these two ITS2 amplicons were from the same DNA sample and tag-primer pairs, but have different PCR cycles (**, 45 cycles for the one in Cell B; *, 35 cycles for the other in Cell C).
