# Supplementary material for: Full-Length Multi-Barcoding: DNA Barcoding from Single Ingredient to Complex Mixtures
Source: Genes (Basel). 2019 May 7;10(5):343. doi: 10.3390/genes10050343 (PMC6562688; doi:10.3390/genes10050343)
Supplement: Supplementary file 1 [file genes-10-00343-s001.zip › Table S3. Species identifying results on Ophiopogon japonicus by FLMB.pdf]

Table S3. Species identifying results on *Ophiopogon japonicus* by FLMB

Sample ID	tag-primer pairs	Nearest match species of resulting reads and its number	
		Barcode of ITS2	Barcode of *psbA-trnH*
MD1	i1**/T1	*Malva verticillata* 98<br>*P. ginseng* 61<br>***O. japonicas* 59**<br>*S. chinensis* 14<br>*Tribulus terrestris* 13<br>uncultured fungus 9<br>*Artemisia tangutica* 7<br>*Cucumis melo* 1<br>*Paeonia suffruticosa* 1<br>*Angelica sinensis* 1<br>*Koelreuteria paniculata* 1<br>Uncultured Hygrocybe 1<br>2 *no-blast-hit*	***O. japonicas* 724**<br>*Panax sp.* 1
MD2	i2**/T2	*P. ginseng* 232<br>*M. verticillata* 109<br>***O. japonicas* 78**<br>*T. terrestris* 53<br>*S. chinensis* 13<br>*Artemisia capillaries* 9<br>*Cimicifuga dahurica* 4<br>*A. sinensis* 2<br>*P. suffruticosa* 1<br>*Sophora japonica* 1<br>Uncultured fungus 3<br>7 *no-blast-hit*	***O. japonicas* 560**<br>*Panax sp.* 14<br>*M. verticillata* 4
MD3<br>(Cell B)	i3**/T3	*T. terrestris* 227<br>*M. verticillata* 210<br>***O. japonicas* 83**<br>*P. ginseng* 30<br>*Juglans regia* 3<br>*S. chinensis* 1<br>14 *no-blast-hit*	***O. japonicas* 736**<br>*Panax sp.* 11<br>*Polygonatum zanlanscianense* 1<br>*T. terrestris* 1
MD4	i4**/T4	*P. ginseng* 107<br>***O. japonicas* 65**<br>*M. verticillata* 63<br>*S. chinensis* 29<br>*T. terrestris*27<br>*A. sinensis* 3<br>8 *no-blast-hit*	***O. japonicas* 784**<br>*Panax sp.* 8<br>*M. verticillata* 1<br>1 *no-blast-hit*
MD3<br>(Cell C)	i3*	***O. japonicas* 127**<br>*M. verticillata* 89<br>*T. terrestris* 72<br>*P. ginseng* 3	/

Note: ** The PCR cycles in the preparation of these four ITS2 amplicons were 35+10; * 35 Cycles. *no-blast-hit*, resulting reads that with low Max. score or low Identities for top hits in BLAST.
