# Supplementary material for: Full-Length Multi-Barcoding: DNA Barcoding from Single Ingredient to Complex Mixtures
Source: Genes (Basel). 2019 May 7;10(5):343. doi: 10.3390/genes10050343 (PMC6562688; doi:10.3390/genes10050343)
Supplement: Supplementary file 1 [file genes-10-00343-s001.zip › Table S4. Validation of different locus between two types of Ophiopogon japonicus in psbA-trnH region.pdf]

Table S4. Validation of different locus between two types of *Ophiopogon japonicus* in *psbA-trnH* region

DNA ID	Original type	Code of tag-primer pairs	genotype and its number in Loc 88 of *psbA-trnH*
MD1	'*Zhe*' *O. japonicus*; *Cixi*, *Zhejiang* province; producing area	T1	721G，3A
MD2	'*Zhe*' *O. japonicus*; *Xiangshan*, *Zhejiang* province; wildness	T2	555G，5A
MD3	'*Chuan*' *O. japonicus*; *Santai*, *Sichuan* province; Market B	T3	726A，10G
MD4	'*Chuan*' *O. japonicus*; *Sichuan* province; Company C	T4	665A，116G，3-
HMA	Mixed by MD1	T17	53G，5A
HMB	Mixed by MD2	T18	20G，3A
HMC	Mixed by MD3	T19	188A，5G
SMS1	'*Chuan*' *O. japonicus* (MD3) as its raw material	T13	130A, 6G
SMS2	'*Chuan*' *O. japonicus* (MD3) as its raw material	T14	161A, 2G
SMS3	'*Chuan*' *O. japonicus* (MD3) as its raw material	T15	28A
